# Higher FT4 or TSH below the normal range are associated with increased risk of dementia: a meta-analysis of 11 studies

**DOI:** 10.1038/srep31975

**Published:** 2016-08-25

**Authors:** Yue Wu, Yuqing Pei, Fei Wang, Danfei Xu, Wei Cui

**Affiliations:** 1 Department of Clinical Laboratory, Peking Union Medical College Hospital, Peking Union Medical College and Chinese Academy of Medical Sciences, Beijing, China

## Abstract

Observational studies of thyroid function and dementia have reported conflicting results. We reviewed cohort and case-control studies from MEDLINE, EMBASE, Web of Science and the Cochrane Library that focused on the association between serum thyroxine, thyrotropin and dementia. A total of 24,952 participants from three case-control and eight cohort studies were included. The relationships between dementia and the per standard deviation (SD) increment of free thyroxine (FT4) (random relative ratio (RR) = 1.08, 95% confidence interval (CI) 1.00–1.17) and thyroid-stimulating hormone (TSH) (fixed RR = 0.91, 95% CI 0.84–0.99) were well established. TSH levels in the low category were associated with an increased risk of dementia (fixed RR = 1.60, 95% CI 1.27–2.00). However, the positive association was confined to TSH levels below the normal range (fixed RR = 1.77, 95% CI 1.31–2.39), not those in the lower tertile of the normal range (fixed RR = 1.39, 95% CI 0.98–1.97). Additionally, dementia was not significantly associated with high TSH levels (fixed RR = 0.99, 95% CI 0.76–1.29). Furthermore, there was no positive association between dementia and the low or high categories of TSH in men. Thus, individuals with higher FT4 levels or those with TSH levels below the normal range have an increased risk of dementia.

Dementia is a clinical syndrome characterized by a cluster of symptoms and signs that results in complex cognitive decline; it has emerged as one of the greatest health threats during old age and has substantial socioeconomic costs. Alzheimer’s disease (AD), which is the most common type of dementia, constitutes 50–70% of cases^1^ and is one of the top 8 major health problems in the world^2^. Despite recent advances in research, major scientific gaps remain in the understanding of the intrinsic mechanism of dementia. In the absence of medications that effectively cure or slow the progression of these diseases, especially AD^3^,^4^, considerable challenges associated with early intervention remain. The development of preventive measures for dementia requires a greater understanding of the risk factors for this disease. Accordingly, we may prevent the elderly from further cognitive decline.

Thyroid hormone (TH) regulates metabolic processes that are essential for normal growth and development as well as neurocognitive function after development^5^. Thyroid-stimulating hormone (TSH) targets the thyroid gland and triggers the release of thyroxine (T4) and triiodothyronine (T3) when stimulated by the hypothalamic secretion of thyrotropin-releasing hormone (TRH)^6^. The relationship between thyroid-related hormones and dementia is controversial, especially regarding TSH (whether outside or within the normal range)^7^,^8^,^9^,^10^. According to laboratory tests, thyroid dysfunction can be classified as overt, with both abnormal TSH and free T4 (FT4) levels, or as subclinical, with abnormal TSH but normal FT4 levels. Some studies have demonstrated a relationship between overt thyroid disease and cognitive disturbances^11^,^12^. However, the influence of subclinical thyroid disease on cognition remains unclear. Since Wahlin *et al.*^13^ first reported an association between poor cognition and low serum TSH levels within the normal range, more researchers have become interested in the impact of thyroid hormone levels (within the normal range) on cognition^7^,^14^. Moreover, the role of FT4 levels in the development or progression of dementia remains controversial. Certain large prospective studies have observed that higher FT4 levels are related to dementia^15^,^16^, whereas other studies have not reported this association^10^,^17^. Therefore, it is necessary to conduct a systematic review and meta-analysis to summarize the published findings and to obtain a better understanding of the association between FT4, TSH and dementia.

## Results

### Eligible studies and characteristics

**Figure 1:**
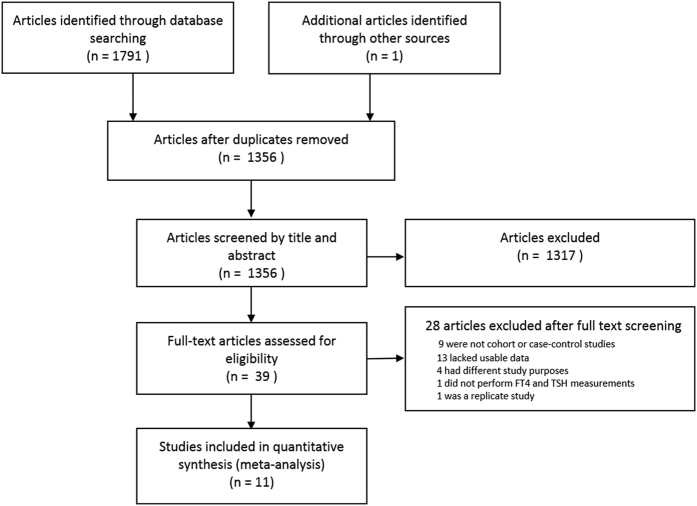
Flow diagram of study retrieval and selection.

The search strategy identified 1791 citations for evaluation. A manual review of the study references yielded one additional study. After carefully scanning the titles and abstracts, 39 articles that were potentially eligible for inclusion were retrieved. After reading the full texts, 28 articles were excluded for the following reasons: nine studies were not cohort or case-control studies^18^,^19^,^20^,^21^,^22^,^23^,^24^,^25^,^26^; 13 lacked usable data^13^,^14^,^17^,^27^,^28^,^29^,^30^,^31^,^32^,^33^,^34^,^35^,^36^; four had different study purposes^37^,^38^,^39^,^40^; one did not perform FT4 and TSH measurements^41^; and one was a replicate study with a shorter follow-up^42^ than that by de Jong *et al.*^10^ (Fig. 1). Eleven studies published from 2003 to 2016 that evaluated a total of 24,952 participants, including 1526 patients with dementia, were finally included. The detailed information is provided in Table 1. Among the included studies, three^43^,^44^,^45^ were case-control studies, and eight^7^,^8^,^9^,^10^,^15^,^16^,^46^,^47^ were cohort studies. Specifically, the work by Tan *et al.*^9^ was analyzed by gender and was treated as two individual studies. Additionally, two studies^15^,^16^ included only men. Most studies controlled for some conventional risk factors, including age (n = 11), gender (n = 11) and thyroid medication (n = 7). Most of the included studies were of high quality and scored from 7 to 9 on the Newcastle Ottawa Scale (NOS). Only one was of moderate quality, with a score of 6 (Supplementary Table 1). The average score was 7.3, and the follow-up duration ranged from 4 to 17 years.

### Meta-analysis

**Figure 2:**
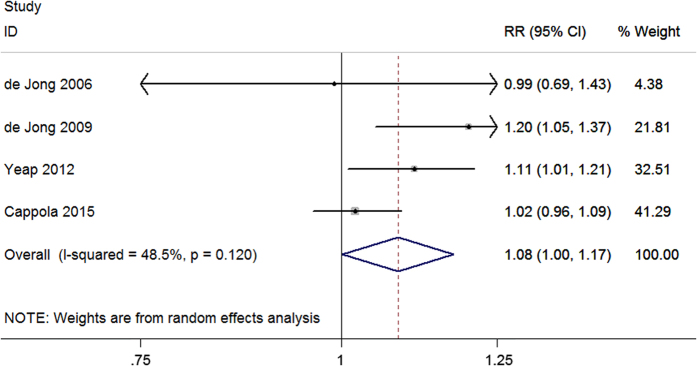
Forest plot of FT4 per SD increment and dementia. The estimated RRs and 95% CIs are plotted with boxes and horizontal lines.

**Figure 3:**
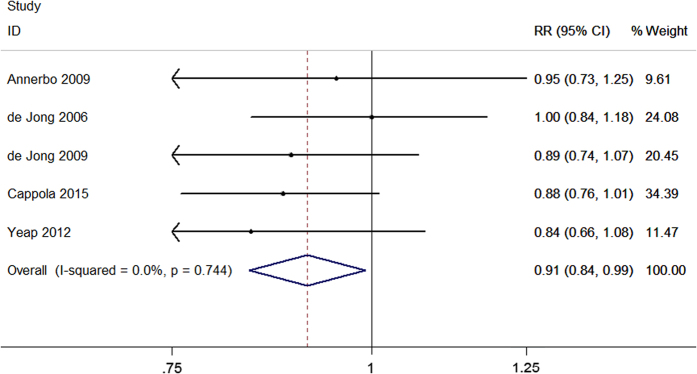
Forest plot of TSH per SD increment and dementia. The estimated RRs and 95% CIs are plotted with boxes and horizontal lines.

**Figure 4:**
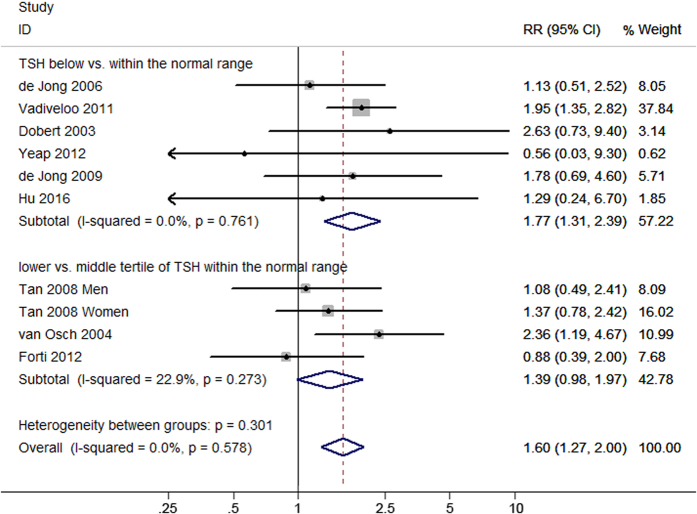
Forest plot of the low vs. middle categories of TSH levels and dementia. Two subgroups were analyzed: TSH levels below vs. within the normal range and the lower vs. middle tertiles of TSH levels within the normal range. The estimated RRs and 95% CIs are plotted with boxes and horizontal lines.

Four studies evaluated the association between serum FT4 concentration and dementia. The pooled results revealed that the per standard deviation (SD) increment of FT4 was associated with an increased risk of dementia (relative ratio (RR) = 1.08, 95% confidence interval (CI) 1.00–1.17) with a heterogeneity (I^2^) of 48.5% (P = 0.120) using the random effect model (Fig. 2). Five studies considered TSH level as a continuous variable to analyze its association with dementia. Meta-analysis using a fixed effect model showed a significant association between the per SD increment of TSH and dementia (fixed RR = 0.91, 95% CI 0.84–0.99; I^2^ = 0%, P = 0.744) (Fig. 3). Moreover, ten studies evaluated the risk estimates of different TSH categories and dementia. When comparing the low and middle categories of TSH levels, the fixed model showed that low TSH levels were significantly associated with an increased risk of dementia (fixed RR = 1.60, 95% CI 1.27–2.00; I^2^ = 0%, P = 0.578) (Fig. 4). Furthermore, the risk estimates for the high and middle categories of TSH levels indicated an inverse but insignificant association between high TSH levels and dementia (fixed RR = 0.99, 95% CI 0.76–1.29; I^2^ = 17.0%, P = 0.291) (Supplementary Figure 1).

### Subgroup analyses

Subgroup analyses were performed on studies with a similar study design (cohort or case-control) and data on TSH levels and gender. Stratified analyses were not performed using the per SD increment of FT4 and TSH levels model because all the included studies were cohort studies. When low and middle categories of TSH levels were compared, the results of analyses stratified by study design were consistent with the overall trend. Additionally, case-control studies showed a positive association between high TSH levels and dementia (fixed RR = 0.54, 95% CI 0.30–1.00; I^2^ = 0%, P = 0.440), while cohort studies did not show this association. For data types regarding TSH levels, there were two low categories: the lower tertile of TSH levels within the normal range and TSH levels below the normal range. Likewise, there were two high categories: the upper tertile of TSH levels within the normal range and TSH levels above the normal range. Subgroup analyses revealed that only TSH levels below the normal range (fixed RR = 1.77, 95% CI 1.31–2.39; I^2^ = 0%, P = 0.761, not those in the lower tertile of the normal range (fixed RR = 1.39, 95% CI 0.98–1.97; I^2^ = 22.9%, P = 0.273) or those in the two high categories, were associated with dementia. Three studies evaluated the relationship between TSH and dementia in men; meta-analyses of the low and high categories of TSH levels showed no association with dementia in men (Supplementary Figure 2). The detailed results are provided in Table 2.

### Sensitivity analysis and publication bias

All of the models demonstrated stability in the sensitivity analysis when each study was individually omitted (Supplementary Figure 3). Visual inspection of the funnel plots for each model did not show asymmetry, except for the model of the per SD increment of FT4 (Supplementary Figure 4); however, Egger’s test found no statistical evidence of publication bias for all models (per SD increment of FT4, P = 0.621; per SD increment of TSH, P = 0.972; low vs. middle categories of TSH levels, P = 0.252; high vs. middle categories of TSH levels, P = 0.480). Funnel plot and Egger’s test were not evaluated for subgroup analyses in men, because the numbers of included studies were small.

## Discussion

To the best of our knowledge, this study is the first meta-analysis assessing the relationship between FT4 and dementia. In the model of per SD increment of FT4 and dementia, we found an association between higher FT4 levels and an increased risk of dementia in community-dwelling elderly from four prospective studies with high quality. Additionally, FT4 was reported to be related to brain atrophy of the hippocampus and amygdala^10^, which is strongly associated with the risk of dementia^48^.

Furthermore, the relationship between the per SD increment of TSH and dementia was well established, showing that lower TSH levels are related to an increased risk of dementia. This finding was supported by the risk estimate of the low category of TSH levels and dementia. Notably, we performed a further subgroup analysis based on the type of data on TSH levels. Interestingly, we found that the positive association between low TSH levels and a high risk of dementia was confined to TSH levels below the normal range, rather than those within the normal range. Due to the limited number of studies on various tertiles of TSH within the normal range, more large studies are urgently needed to obtain a comprehensive and precise conclusion.

In addition, dementia was not significantly associated with TSH levels in the high categories (above the normal range or in the upper tertile within the normal range). Stratified analysis by case-control study showed high TSH levels had a protective tendency for dementia, while the analysis by cohort study showed no association between high TSH levels and dementia, indicating that this association still needs to be investigated. Our observation was also consistent with the two placebo-controlled, double-blind randomized clinical trials of T4 medication administered for 1 year^49^,^50^. The two studies both failed to show the effect of T4 replacement therapy on improving cognitive function.

Furthermore, a differential risk by gender in the TSH analyses existed in several studies^9^,^36^,^51^. These studies collectively indicated that women were more susceptible to the effects of TSH on cognition. In our study, the result of the subgroup analysis of gender in three studies showed no relationship between TSH and dementia in men in the high or low categories of TSH levels; however, considering the limited number of studies, caution should be taken when concluding a negative association; additional well-designed studies stratified by gender should be conducted.

Remarkably, some scholars have suggested that low TSH and high FT4 levels may be biomarkers of age, and both parameters are associated with other diseases that occur in patients of advanced age, including dementia. In addition, low TSH levels might be a consequence of AD-related neurodegeneration, which could reduce the secretion of TRH by the hypothalamus or decrease the responsiveness of the pituitary gland to TRH^52^. Moreover, other studies have demonstrated a relationship between thyroid hormone and amyloid, which has an essential role in AD development. These findings raise the question of whether low TSH and high FT4 levels are contributors to, rather than biomarkers or even consequences of, poor cognitive outcomes in older adults. However, our study explored positive associations between higher FT4 levels and TSH levels below the normal range and dementia, and a lack of association between high TSH levels and dementia was noted.

Our meta-analysis had some limitations. First, although bias may exist due to the observational nature of the included studies and different data collection methods (prospective or retrospective), the relatively large combined sample size and good quality of the included studies potentially strengthen the power of the analysis. Second, the methods used to detect thyroid function and covariates differed among the studies. No specific reference range of hormone levels was provided. Moreover, three RRs in the TSH model were calculated manually based on the available data in the respective studies, whereas other data were extracted from the most adjusted regression models. A more precise analysis should be conducted when individual data are available. Third, a subgroup analysis by age was not performed because of insufficient data. The incidence of AD differs with age, and the prevalence doubles every five years after the age of 60^53^. Fourth, the prevalence of high TSH concentrations is higher in individuals of Caucasian descent than in those of African descent; this difference indicates a genetic influence on TSH secretion^54^. Most individuals included in our study were Caucasians. Thus, a subgroup analysis by race was not conducted.

In conclusion, this report is the first meta-analysis illustrating the contribution of TSH and FT4 to dementia. Based on 24,952 participants including 1526 patients with dementia, an increased risk of dementia in individuals with higher serum FT4 levels and in those with TSH levels below the normal range was found. The present study implied T4 replacement therapy may be administered cautiously, as no specific relationship was shown between high TSH levels and dementia. Moreover, individuals receiving T4 replacement therapy may develop a low or even a suppressed TSH level^55^, which could consequently lead to an increased risk of dementia. However, additional large, prospective, cohort studies and randomized controlled trials are still needed to determine the causal association between thyroid hormones and dementia.

## Methods

This meta-analysis was conducted according to the guidelines of the Preferred Reporting Items for Systematic Reviews and Meta-Analyses (PRISMA) 2009 Checklist (http://www.prismastatement.org/statement.htm) and the Meta-analysis of Observational Studies in Epidemiology group (MOOSE)^56^.

### Search strategy and study selection

We searched Medline, Embase, Web of Science and the Cochrane Library for all published studies up to June 7, 2016, that evaluated the relationship between thyroid function and cognitive decline without any restrictions. The key search terms were “thyroxine (T4)”, “thyrotropin (TSH)”, “triiodothyronine (T3)”, “hyperthyroidism”, “hypothyroidism”, “euthyroidism”, “subclinical hyperthyroidism”, “subclinical hypothyroidism”, “thyroid dysfunction or disorders”, “dementia”, “Alzheimer’s disease (AD)”, “cognitive decline or impairment or defect”, “mild cognitive impairment (MCI)” and “cohort or case-control or longitudinal or follow-up”. Supplementary Figure 5 contains the MEDLINE search terms. We also manually searched the bibliographies of all qualified studies for other potential articles. Wu Y and Pei Y independently evaluated the retrieved studies by thoroughly reading the titles and abstracts with pre-designed inclusion criteria. Full texts were further checked if necessary. If the required data were not reported in a potential article, we contacted the corresponding author for more details. Any disagreement between the two investigators was resolved by consensus or by discussion with a third reviewer, Wang F.

Studies were initially included in the detailed assessment if they met the following criteria: 1) sufficient information on the study population; 2) cohort or case-control studies; 3) clear criteria for the outcome (dementia or AD) diagnosed according to internationally accepted criteria for dementia (DSM-III-R^57^ or DSM-IV^58^) and AD (NINCDS-ADRDA^59^), by a multi-disciplinary team (neurologists, psychiatrists, and psychologists) or by record linkage; 4) sufficient data on risk estimates of the association between FT4 or TSH levels and dementia or AD: RR, hazard ratio (HR), or odds ratio (OR) with 95% CIs or the number of outcome events; 5) adjustments for potential confounders; and 6) valid measurements of FT4 and TSH. We excluded studies that were cross-sectional, lacked usable data, or focused on vascular dementia or dementia secondary to other diseases. If multiple reports used the same population, the study with the longest follow-up was included. Reviews, case reports, abstracts and conference proceedings were excluded.

### Data extraction and quality assessment

Two investigators (Wu Y and Pei Y) independently extracted the data from the eligible studies with a common form. The outcome was dementia or AD. The following data were extracted: first author, publication year, study type, country or race, study size, age of participants, gender distribution, duration of follow-up, covariates, and results (Table 1). Generally, the levels of TSH can be divided into three categories: TSH below the normal range, TSH within the normal range, and TSH above the normal range. While in the normal range, TSH levels can be further divided into three tertiles: lower, middle and upper. Thus, the overall TSH levels were classified into three categories in our meta-analysis models: TSH below the normal range or the lower tertile within the normal range, denoted as low; TSH within the normal range or the middle tertile within the normal range, denoted as middle; and TSH above the normal range or the upper tertile within the normal range, denoted as high. Because the data regarding thyroid function varied among the studies, we extracted the following information for the meta-analysis: 1) risk estimates per SD increment of FT4/TSH levels; 2) risk estimates for TSH levels below or above the normal range compared with TSH levels within the normal range; and 3) risk estimates for lower or upper tertiles compared with the middle tertile of TSH levels within the normal range. Of the studies that reported risk estimates with multiple-level adjustments, we included those that adjusted for the most covariates.

The Newcastle Ottawa Scale, a measure of the quality of observational studies, was used to assess the quality of the selected studies. This scale consists of three domains, including the selection of cases, comparability of populations, and ascertainment of exposure. Scores of 0–3, 4–6, and 7–9 indicate low, moderate, and high quality, respectively. Two investigators independently performed the quality assessment, and disagreements were resolved by discussion.

### Data synthesis and statistical analyses

Dichotomous data were calculated as the OR and 95% CI. In the meta-analysis, the RRs were used for risk estimates. ORs and HRs were also treated as RRs. Heterogeneity between studies was assessed using the I^2^ statistic: I^*2*^ < 25%, low; between 25 and 50%, moderate; and >50%, high^60^. If moderate or high heterogeneity was found, a random effect model was used^61^; otherwise, the fixed effect model was applied^62^. Subgroup analyses based on study design, data type regarding TSH levels and gender were performed to explore the sources of heterogeneity. To assess the stability of the results, a one-way sensitivity analysis was performed by omitting each study in turn from the analysis. Publication bias was assessed by funnel plots and Egger’s linear regression test^63^. All analyses were conducted using STATA 12.0 (StataCorp, College Station, TX, USA), and a two-sided P < 0.05 was considered significant, except for Egger’s test, in which the P value was set at 0.10 to be more conservative.

## Additional Information

**How to cite this article**: Wu, Y. *et al.* Higher FT4 or TSH below the normal range are associated with increased risk of dementia: a meta-analysis of 11 studies. *Sci. Rep.* **6**, 31975; doi: 10.1038/srep31975 (2016).

## Supplementary Material

Supplementary Information

## Figures and Tables

**Table 1:** Characteristics of included case-control and cohort studies.

First author	Country (race)	Study size (females, %)	Mean age (range)	Follow up (years)	Covariates	Results	NOS score
Case-control studies							
Dobert 2003^43^	Germany (Caucasian)	77 dementia (46.8%); 42 controls (47.6%)	AD, 69.8 Control, 63.9	—	Age, sex, thyroid mediation	Positive association between lower TSH and dementia.	7
van Osch 2004^44^	UK (Caucasian)	178 AD (43.0%); 291 controls (48.0%)	73.7	—	Age, sex, education, APOEε4, Hcy, depression, Cr, thyroid mediation	Positive association between lower TSH and AD.	7
Hu 2016^45^	China (Asian)	154 AD (46.8%); 77 controls (44.2%)	AD, 63.5 Controls, 64.1	—	Age, sex, education, BMI	Negative association between AD and hypothyroidism and SH.	6
Cohort studies							
de Jong 2006^10^	Netherlands (Caucasian)	1,025 communities (51.2%)	72.3 (60–90)	5.5	Age, sex, education, smoking, Hcy depression, smoking, Cr, APOEε4, DM, AF, BMI, medication use, cholesterol,	Negative association between TSH/FT4 and dementia.	9
Tan 2008^9^	USA (Caucasian)	1864 communities (59.0%)	71	12.7	Age, stroke, education, Hcy, BMI, AF	Positive association between low/high TSH and AD in women.	7
de Jong^16^	Hawaii (Japanese -American)	665 communities (0%)	77.3–78.6 (71–93)	4.7	Age, education, depression, albumin, BMI, TC, HDL-C, DM, smoking, thyroid mediation, SBP, DBP	Positive association between higher FT4 and dementia.	7
Annerbo 2009^46^	Sweden (Caucasian)	200 communities (79.5%)	81.0 (75–93)	6.7	Age, sex, education	Negative association between TSH and dementia.	7
Forti 2011^8^	Italy (Asian)	660 communities (52.9%)	73.3 (65–91)	4	Age, gender, education, BMI, hypertension, cholesterol, Hcy, DM, CVD, GDS	Negative association between TSH and AD.	7
Vadiveloo 2011^47^	UK (Caucasian)	12,115 communities (NR)	66.5	5.6	Age, gender, history of dementia and psychiatric disorders, thyroid medication	Positive association between SH and dementia.	7
Yeap 2012^15^	Australia (Caucasian)	3401 communities (0%)	(70–89)	5.9 (median)	Age, BMI, smoking, education, DM, CVD, hypertension, MMSE, social support, sensorial impairment, thyroid medication	Positive association between higher FT4 and dementia in men.	9
Cappola 2015^7^	USA (African18.6%, Caucasian 81.4%)	2843 communities (56.2%)	74.5	17	Age, sex, race, thyroid medication, hypertension, DM, current smoking, alcohol, BMI, TC, stroke, APOEε4, claudication	Positive association between higher TSH and lower incidence of dementia.	7

Key: NOS: Newcastle Ottawa Scale AD, Alzheimer’s disease; Hcy, homocysteine; BMI, body mass index; SH: subclinical hyperthyroidism; Cr, creatinine; DM, diabetes; AF, atrial fibrillation; TC, total cholesterol; HDL-C, high density lipoprotein cholesterol; SBP, systolic blood pressure; DBP, diastolic blood pressure; CVD, cardiovascular disease; GDS, Geriatric Depression Scale score; NR, not reported; MMSE, minimum mental state examination.

**Table 2:** Stratified analysis of the association between TSH and dementia.

Subgroup	N	Risk estimate		Heterogeneity	
		Pooled RR (95% CI)	P_1_	I^2^	P_2_
TSH: low category vs. middle category	10	1.6 (1.27, 2.00)	<0.001	0.00	0.58
Study type					
Cohort	7	1.5 (1.17, 1.92)	0.001	0.00	0.49
Case-control	3	2.25 (1.28, 3.96)	0.005	0.00	0.78
Data type					
TSH below vs. within the normal range	6	1.77 (1.31, 2.39)	<0.001	0.00	0.76
Lower vs. middle tertile of TSH within the normal range	4	1.39 (0.98, 1.97)	0.062	0.23	0.27
TSH: high category vs. middle category	9	0.99 (0.76, 1.29)	0.923	0.17	0.29
Study type					
Cohort	6	1.14 (0.85, 1.54)	0.385	0.00	0.65
Case-control	3	0.54 (0.30, 1.00)	0.048	0.00	0.44
Data type					
TSH above vs. within the normal range	6	0.91 (0.61, 1.34)	0.619	0.00	0.77
Upper vs. middle tertile of TSH within the normal range	3	1.07 (0.74, 1.54)	0.734	0.70	0.03
Gender: male					
Low category vs. middle category of TSH	3	1.28 (0.70, 2.32)	0.419	0.00	0.62
High category vs. middle category of TSH	3	0.92 (0.60, 1.43)	0.722	0.00	0.57

Key: N, number of studies. P_1_ is an evaluation of the statistical significance level of the risk estimate, while P_2_ is an evaluation of the heterogeneity among included studies. Subgroup analyses were performed by study design (cohort or case-control) and data types of TSH levels. Three studies evaluating the relationship between TSH and dementia in men were analyzed separately.
