# Supplementary material for: Higher FT4 or TSH below the normal range are associated with increased risk of dementia: a meta-analysis of 11 studies
Source: Sci Rep. 2016 Aug 25;6:31975. doi: 10.1038/srep31975 (PMC4997309; doi:10.1038/srep31975)
Supplement: Supplementary Information [file srep31975-s1.pdf]

Higher FT4 or TSH below the normal range are associated with increased risk of dementia: a meta-analysis of 11 studies

Yue Wu[1], Yuqing Pei[1], Fei Wang[1], Danfei Xu[1] and Wei Cui[1]*

[1] Department of Clinical Laboratory, Peking Union Medical College Hospital, Peking Union Medical College and Chinese Academy of Medical Sciences, Beijing, China

* *Corresponding author e-mail:* cuiw@pumch.cn

Supplementary Table 1 Quality assessment of included studies by Newcastle-Ottawa scale

Reference	Selection				Comparability	Outcome			
cohort study	*Representative of cases*	*Selection of controls*	*Ascertainment of exposure*	*Outcomes present at start of study*	*Comparability the design or analysis*	*Assessment of outcome*	*Adequate follow-up time （≧ 5 year）*	*Adequacy of follow up (≧ 80%)*	**Overall Quality**
de Jong 2006	★	★	★	★	★★	★	★	★	9
Tan 2008	★	★	★	★	★	★	★	–	7
de Jong 2009	★	★	★	★	★★	★	–	–	7
Annerbo 2009	★	★	★	★	★	★	★	–	7
Forti 2011	★	★	★	★	★	★	–	★	7
Vadiveloo 2011	★	★	★	–	★★	★	★	–	7
Yeap 2012	★	★	★	★	★★	★	★	★	9
Cappola 2015	★	★	★	–	★★	★	★	–	7
case-control study	*Case Definition*	*Representative of Cases*	*Selection of Control*	*Definition of Control*	*Comparability the design or analysis*	*Ascertainment of exposure*	*Same Method*	*Non - Response Rate*	**Overall Quality**
Dobert 2003	★	★	–	★	★★	★	★	–	7
van Osch 2004	★	–	★	★	★★	★	★	–	7
Hu 2006	★	–	★	★	★	★	★	–	6

Supplementary Figure legends

Supplementary Figure 1 Forest plot of high vs. middle category of TSH levels with dementia.

Supplementary Figure 2 Forest plot of low vs. middle category of TSH levels with dementia and high vs. middle category of TSH levels with dementia in men.

Supplementary Figure 3 Sensitivity analysis of all models.

Supplementary Figure 4 Funnel plots to assess the risk of publication bias.

Supplementary Figure 5: MEDLINE search strategy

Supplementary Figure 1: Forest plot of high vs. middle category of TSH levels with dementia. Two subgroups were analyzed which were TSH above vs. within the normal range and upper vs. middle tertile of TSH within the normal range. The estimate of RRs and their 95% CIs are plotted with boxes and horizontal lines.

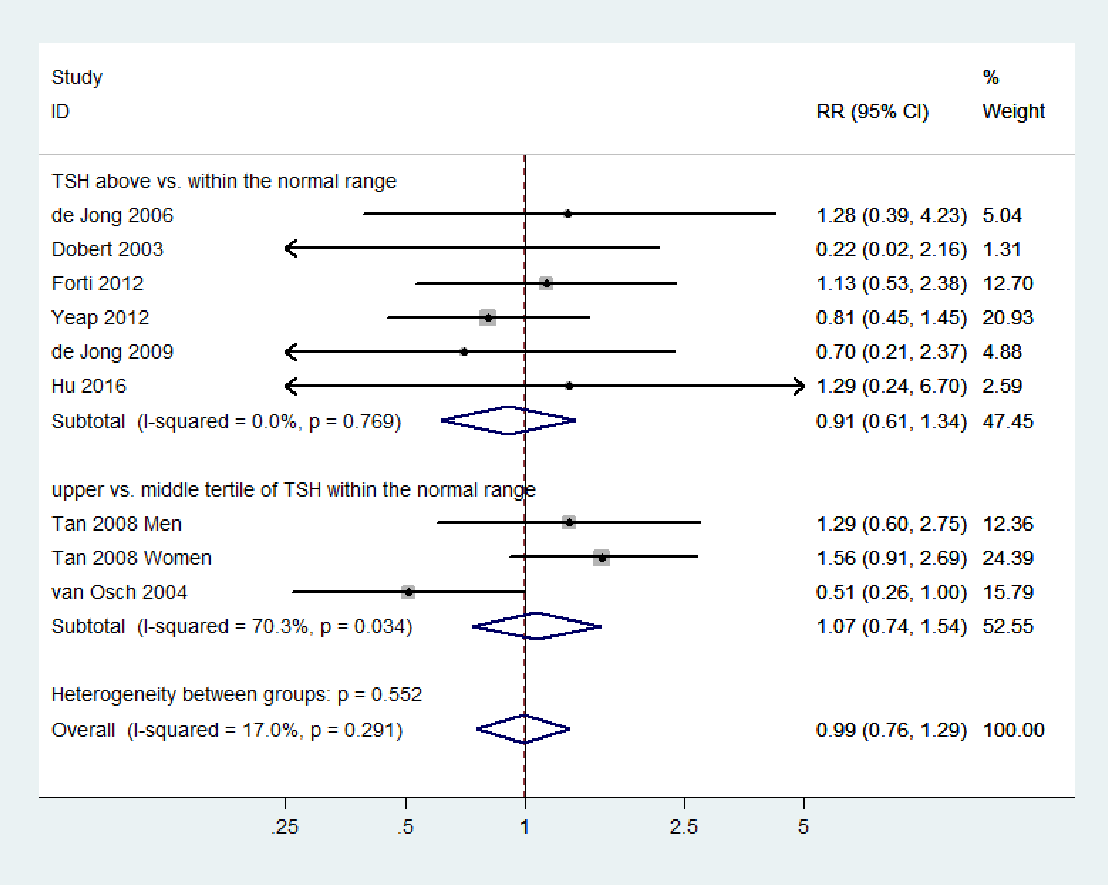

Supplementary Figure 2: Forest plot of low vs. middle category of TSH levels with dementia and high vs. middle category of TSH levels with dementia in men. The estimate of RRs and their 95% CIs are plotted with boxes and horizontal lines.

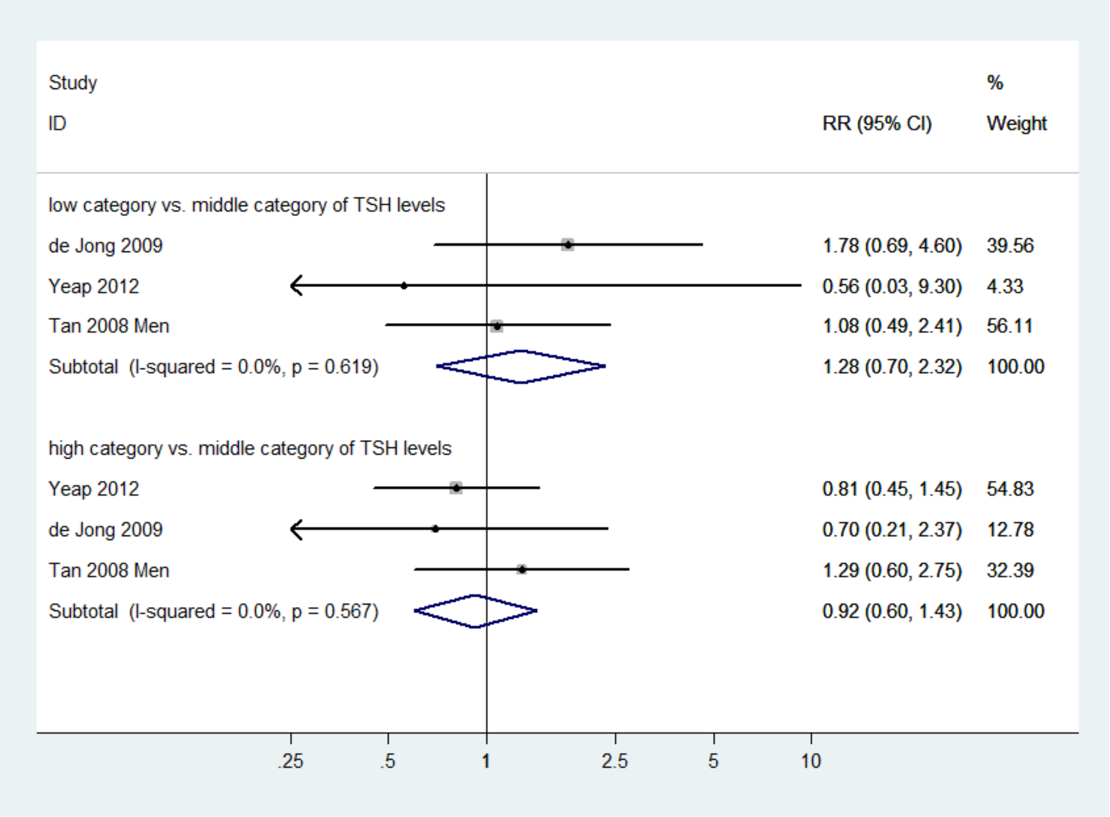

Supplementary Figure 3: Sensitivity analysis of all models. A: per SD increment of FT4 and dementia B: per SD increment of TSH and dementia C: low vs. middle categories of TSH levels and dementia D: high vs. middle categories of TSH levels and dementia E: low vs. middle categories of TSH levels and dementia in men F: high vs. middle categories of TSH levels and dementia in men.

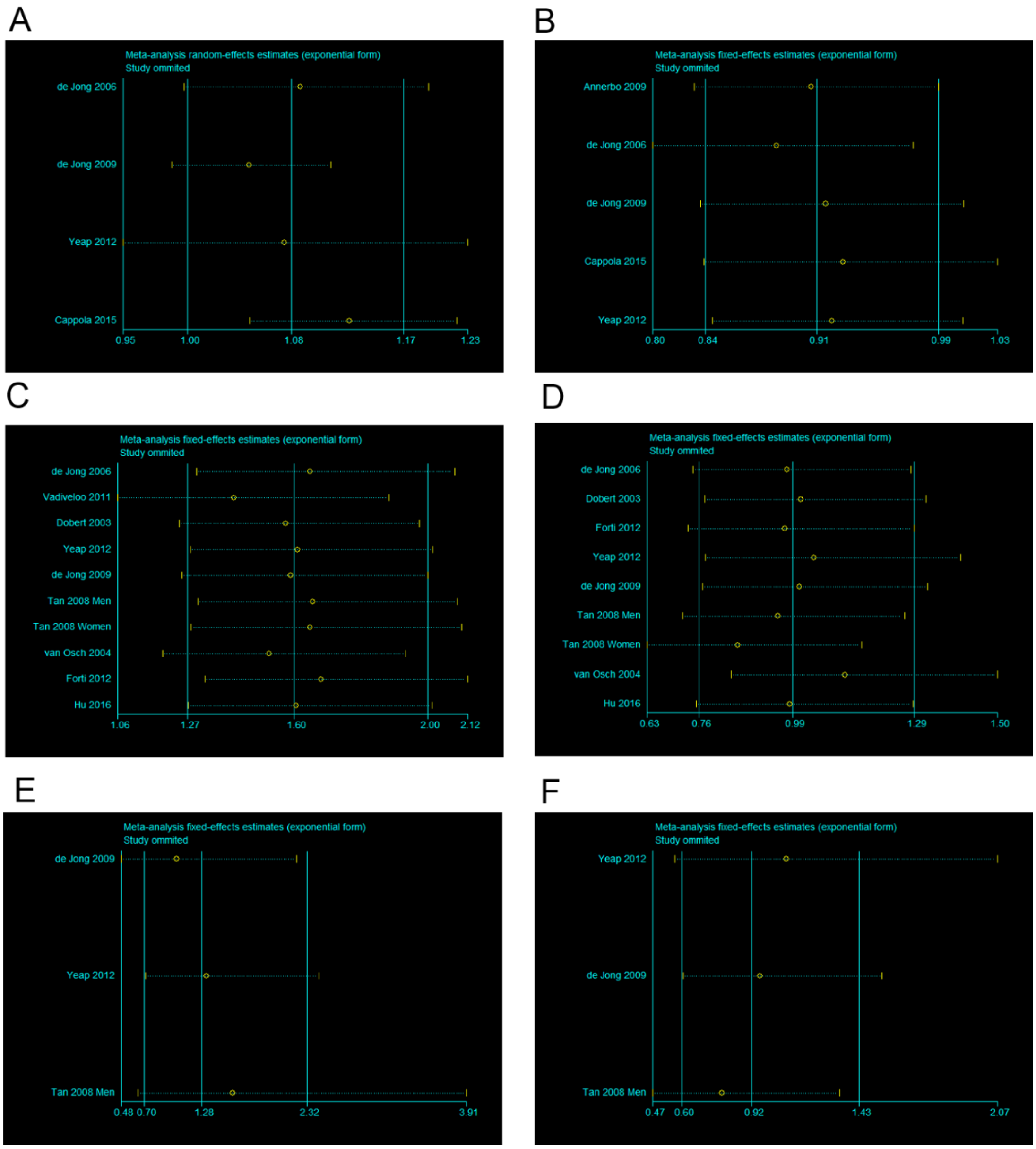

Supplementary Figure 4: Funnel plots to assess the risk of publication bias.

A: per SD increment of FT4 and dementia B: per SD increment of TSH and dementia C: low vs. middle categories of TSH levels and dementia D: high vs. middle categories of TSH levels and dementia

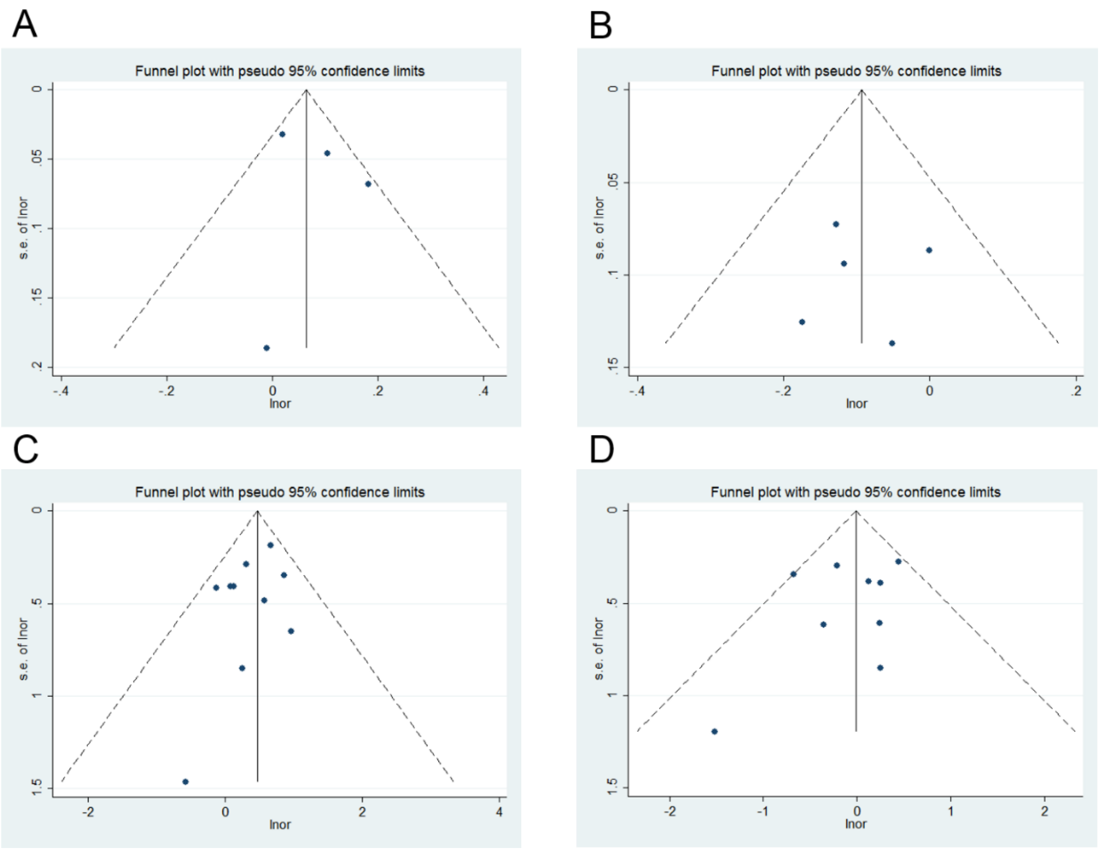

Supplementary Figure 5: MEDLINE search strategy

1 exp Cognition Disorders/ or exp Alzheimer Disease/ or exp Mild Cognitive Impairment/ or exp Dementia/ or exp Memory Disorders/

2 (mci or ad).ti,ab,kw.

3 (dementia or "alzheimer* disease" or "cognitive dysfunction" or "cognitive decline" or "cognitive impairment" or "cognitive defect" or "cognitive disorder*" or "memory disorder*").mp.

4 or/1-3

5 exp Hypothyroidism/ or exp Thyroxine/ or exp Triiodothyronine/ or exp Hyperthyroidism/ or exp Thyrotropin/

6 ("Thyroid Stimulating Hormone*" or thyrotropin or triiodothyronine or "thyroid hormone*" or "subclinical hypothyroidism" or hypothyroidism or "subclinical hyperthyroidism" or hyperthyroidism or euthyroid).mp.

7 (tsh or ft3 or ft4 or tt4 or tt3 or "total T3" or "total T4").ab,kw,ti.

8 or/5-7

9 4 and 8

10 exp cohort studies/

11 cohort$.tw.

12 controlled clinical trial.pt.

13 epidemiologic methods/

14 limit 13 to yr=1966-1989

15 exp case-control studies/

16 (case$ and control$).tw.

17 or/10-12,14-16

18 9 and 17
